# How Viruses Hijack and Modify the Secretory Transport Pathway

**DOI:** 10.3390/cells10102535

**Published:** 2021-09-24

**Authors:** Zubaida Hassan, Nilima Dinesh Kumar, Fulvio Reggiori, Gulfaraz Khan

**Affiliations:** 1 Department of Medical Microbiology and Immunology, College of Medicine and Health Sciences, United Arab Emirates University, Al Ain P.O. Box 17666, United Arab Emirates; zubaidahassan@mautech.edu.ng; 2 Department of Microbiology, School of Life Sciences, Modibbo Adama University, Yola PMB 2076, Nigeria; 3 Department of Biomedical Sciences of Cells and Systems, University of Groningen, University Medical Center Groningen, 9713 AV Groningen, The Netherlands; n.dinesh.kumar@umcg.nl (N.D.K.); f.m.reggiori@umcg.nl (F.R.); 4 Department of Medical Microbiology and Infection Prevention, University of Groningen, University Medical Center Groningen, 9713 AV Groningen, The Netherlands

**Keywords:** endoplasmic reticulum, Golgi, viruses, plasma membrane, intracellular trafficking, vesicles, membrane rearrangements

## Abstract

Eukaryotic cells contain dynamic membrane-bound organelles that are constantly remodeled in response to physiological and environmental cues. Key organelles are the endoplasmic reticulum, the Golgi apparatus and the plasma membrane, which are interconnected by vesicular traffic through the secretory transport route. Numerous viruses, especially enveloped viruses, use and modify compartments of the secretory pathway to promote their replication, assembly and cell egression by hijacking the host cell machinery. In some cases, the subversion mechanism has been uncovered. In this review, we summarize our current understanding of how the secretory pathway is subverted and exploited by viruses belonging to *Picornaviridae, Coronaviridae*, *Flaviviridae,* *Poxviridae*, *Parvoviridae* and *Herpesviridae* families.

## 1. Introduction

Eukaryotic cells have numerous compartments to carry out specialized functions. These subcellular organelles are separated from each other, and from the cell’s cytoplasm, by membranes. These compartments are nevertheless interconnected and communicate via intricate mechanisms to coordinate cellular functions. One of these mechanisms is vesicular transport [1]. The process of vesicular transport consists of vesicle budding at the donor compartment, intracellular movement of the vesicle and vesicle docking and fusion with the acceptor compartment. The budding of cargo-loaded vesicles at the donor compartments is aided by specific cargo receptors; adaptor proteins; GTPases; and coat proteins, such as the coatomer protein complex I (COPI) and COPII [1,2]. The fusion of transport vesicles with the correct acceptor compartment, in contrast, is assured and mediated by the RAS superfamily of small G proteins (RAB) GTPases, tethering factors and soluble *N*-ethylmaleimide-sensitive attachment receptors (SNAREs) [1,2,3]. The cytoskeleton and motor proteins also play an important role in this latter event, especially when the acceptor compartment is at a distance from the donor one [4,5,6].

Upon infection, viruses hijack cellular pathways to promote their propagation. In addition to the genes that encode for viral structural components, viruses also express proteins that are not incorporated into the progeny virions but are essential for the viral life cycle [7]. These proteins modify intracellular compartments to generate new membranous structures to carry essential functions, such as virus replication, assembly and egression. In response to infection, cells also modify their normal vesicular transport pathways to defend themselves from the infection [8,9]. For example, pattern-recognition receptors detect, compartmentalize and stimulate phagocytosis [10]. The induction of autophagy and the formation of aggresomes are other mechanisms by which cells can recognize and degrade viral components [11,12]. Similarly, intrinsic antiviral factors induced following infection can selectively and immediately block the replication of some viruses [13]. Viruses, however, have evolved equally elegant mechanisms to circumvent these antiviral defenses, such as hijacking the cellular RAB proteins [14]. RAB7 is an essential regulator of the endolysosomal system. In hepatitis C virus (HCV) and vaccinia virus-infected cells, it has been shown that the RAB-interacting lysosomal protein (RILP) is modified, thereby disrupting RAB7–RILP interaction. This in turn, prevents lysosomal degradation of the virus-containing vesicles and promotes virion secretion [15,16]. Similarly, the intracellular trafficking and morphogenesis of human cytomegalovirus (CMV), a member of the *Herpesviridae* family, depend on RAB6, which relocalizes from the perinuclear space to the viral particle assembly sites at the trans-Golgi network (TGN) [17]. Other viruses, including picornaviruses, hijack the secretory pathway via lipid kinase phosphatidylinositol 4-kinase (PI4K) III [18,19]. Class III PI4K is a Golgi lipid kinase important for Golgi structure and function, and it activates lipid kinases [18,19]. Viruses usurp this pathway for a supply of essential lipid to the viral replication platforms; this in turn depletes lipid from the host cell [19].

**Figure 1:**
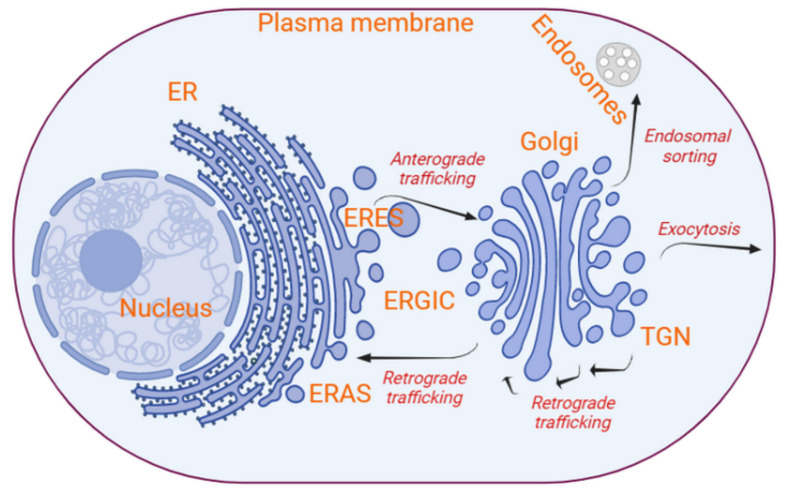
Overview of the secretory transport route and its key components. The compartments characterizing the conventional secretory route are the ER, the ERGIC, the Golgi apparatus (which is subdivided into cis-, medial and trans-Golgi and TGN) and the plasma membrane. Lipid bilayers and proteins are mainly trafficked between these compartments mostly by bidirectional vesicle transport. At a specialized subdomain of the ER, the ERES, cargoes are packed into COPII-coated vesicles through a process that involves SAR1 GTPases and membrane cargo receptors. Upon uncoating, the COPII-coated vesicles fuse with the ERGIC. The ERGIC develops into the cis-Golgi, which then matures into trans-Golgi and finally into the TGN, through a process counterbalanced by the correct relocalization of resident proteins in COPI-coated vesicle-mediated retrograde traffic. The assembly of COPI-coated vesicles is initiated by small GTPases from the ARF protein family and cargo receptors, which promote the incorporation of specific cargo proteins. The TGN is practically a trans-Golgi cisterna, which, through the action of vesicle protein coats and adaptor proteins, vesiculate in an orchestrated manner to generate vesicles with specific cargoes destined to the plasma membrane or the compartments of the endosomal system. In particular, secretory vesicles are characterized by cargo proteins destined to the plasma membrane and extracellular milieu. Vesicle fusion with the acceptor compartments requires tethering factors, RAB GTPases and SNAREs (image created in Biorender).

One of the principal transport routes in eukaryotic cells is the secretory pathway. Newly synthesized proteins and lipids are transported from the endoplasmic reticulum (ER) to the plasma membrane via the ER–Golgi intermediate compartment (ERGIC) and the Golgi apparatus (Figure 1) [6]. While transmembrane proteins and lipids become an integral part of the plasma membrane, soluble proteins are secreted to the extracellular milieu. At the Golgi, a subset of proteins and specific lipids are sorted and delivered to the compartments of the endolysosomal system [20]. In cells infected by specific viruses, particularly RNA viruses, the ER, the ERGIC and/or the Golgi are modified to form structures that are not otherwise present in uninfected cells [21]. These structures are associated with viral replication, assembly and/or egression [7,21]. In this review, we discuss how viruses hijack the secretory pathway and undergo membrane rearrangements for their life cycle. We focus on examples from three families of RNA viruses, namely, *Picornaviridae*, *Coronaviridae* and *Flaviviridae*, and three families of DNA viruses, namely, *Poxviridae*, *Parvoviridae* and *Herpesviridae*.

## 2. The Organization and Dynamics of the Secretory Pathway

The secretory pathway of eukaryotic cells is characterized by the sequential transport of proteins and lipids from the ER to the plasma membrane, via the ERGIC and the Golgi, by vesicles (Figure 1) [22]. Direct transport pathways from the ER to the plasma membrane have also been described, and these types of routes are often defined as unconventional secretion [23,24,25,26]. Vesicle-mediated intracellular transport between subcellular compartments is guided by proteins, such as cargo receptors, small GTPases, vesicle protein coats, tethering factors and SNAREs [14,27,28].

RAB proteins are a large group of proteins belonging to the RAS superfamily of small G proteins and possess GTPase activity. More than five dozen have been identified to date in humans, and most of them are associated with intracellular transport and the secretion of vesicles [14,28]. RAB proteins do not appear to overlap in their function [14,28], and this correlates with the fact that, together with tethering factors and SNAREs, they provide specificity to the vesicular transport; i.e., they guarantee that determined vesicles fuse with the correct acceptor compartment.

Tethering factors are essential molecules that facilitate the docking and fusion of vesicles with their target membrane [29,30]. Tethering factors are subdivided into two groups: homodimers, which form elongated coiled-coil tethers, and hetero-oligomers, multi-subunit tethering complexes, which assemble into more compact tethers. For example, membrane tethering factors and other essential cytosolic components, such as SEC1-MUNC18 (SM) proteins and the *N*-ethylmaleimide-sensitive fusion factor (NSF), bind to SNAREs and regulate their assembly, thereby ensuring the specificity of the vesicle to its target membrane [29,30]. After vesicle binding, the pre-bundled SNAREs (see below) are dismantled by the action of NSF and soluble NSF attachment proteins (SNAPs) and are ready for a new fusion event [30].

SNAREs are evolutionarily conserved proteins that coordinate and orchestrate vesicle formation, trafficking and fusion [30,31,32]. Most SNAREs are composed of a 60–70 residue motif that folds into an amphipathic α-helix motif, known as the SNARE motif, which often protrudes from a C-terminal transmembrane segment [29]. Several of them also have regulatory *N*-terminal domains that mediate their assembly and interaction with other fusion elements during the docking and/or fusion of the vesicles [29]. SNAREs are subcategorized into two groups: target SNAREs (t-SNAREs) and vesicle SNAREs (v-SNAREs). t-SNAREs, also known as Q-SNAREs, are on the acceptor compartment and provide three α-helixes for the fusogenic bundle. Vesicle SNAREs (v-SNAREs), also known as R-SNAREs, are individually present in the transport vesicles. SNARE proteins contain one or two α- helixes, and, generally, four of them (three from the t-SNAREs and one from the v-SNARE) form a highly stable twisted and parallel α-helical bundle that approaches membranes and releases the energy required for their fusion [32,33,34]. These coiled coils of α-helices that lead to the formation of trans-SNARE complexes are also called SNAREpins. The fusion starts at the *N*-terminal ends of the SNARE motifs and propagates toward the C-terminal transmembrane domains [35]. Steric–electrostatic interactions among several SNAREpins make them form a circular cluster at a specific site, known as the fusion pore [36]. Entropic forces within the fusion pore pull the vesicles into the acceptor compartment provoking fusion [35].

### 2.1. Vesicle Formation and Budding at the ER, and Fusion with the ERGIC/Golgi

Different protein coats characterize vesicles destined to the different compartments of the secretory system. COPII-coated vesicles mediate the anterograde transport from the ER to the ERGIC (Figure 1). The ERGIC matures in successive steps into cis-, medial and trans-Golgi and then the TGN through a process that is counterbalanced by the COPI-coated vesicle-mediated retrograde transport of proteins back to the ER or an earlier Golgi cisternae [1,2,8,18,22] (Figure 1). COPI-coated vesicles also appear to be involved in the anterograde transport of big cargo proteins, such as collagen, within Golgi cisternae [37,38]. At the ER, integral membrane proteins and soluble cargo proteins bound to their transmembrane cargo receptors trigger the activation of the small GTPase SAR1 via the transmembrane guanosine exchange factor (GEF) SEC12 [39,40]. Activated SAR1 induces the sequential recruitment of two heterodimeric complexes, SEC23–SEC24 and SEC13–SEC31 [14,27,41]. The SEC23–SEC24 complex selects and binds to the transported integral proteins and loaded cargo receptors. This interaction leads to the formation of complex ternary structures that concentrate the cargo and bends membranes, while SEC13–SEC31 complexes envelope these membrane deformations creating a COPII-coated vesicle structure that is released by fission from the ER [27]. The COPII-coated vesicles are mainly formed at the ER exit sites (ERESs) (Figure 1). ERESs are specialized long-lived subdomains of the ER that link secretory proteins to COPII-coated vesicles. This link is mediated by the SEC16-positive macro-subdomains [42]. In particular, SEC16 bridges the COPII coat inner protein layer, i.e., SAR1 and SEC23–SEC24, to the cargo proteins [42].

The released COPII-coated vesicles travel to the ERGIC and then to the cis-Golgi guided by specific RAB GTPases, particularly RAB1, which, along with the extended coiled-coil domain tethers, such as p115, facilitate the correct targeting of COPII-coated vesicles [42]. Other tethering factors include the *cis*-Golgi matrix of 130 kDa (GM130), the Golgi reassembly stacking protein of 65 kDa (GRASP65) and the transport protein particle I (TRAPPI) complex, which act as a GEF for RAB1. Once activated on an acceptor membrane, RAB1 generates a localized signal that tethers COPII vesicles [42,43]. A subunit of the TRAPPI complex, BET3, binds to the COPII coat via SEC23 and brings the vesicle closer to the Golgi membrane [43]. Alternatively, a homodimer coiled-coil tethering factor, golgin, attracts COPII-coated vesicles by binding them via its C-terminus and/or its RAB GTPase domain, anchoring the vesicles to the *cis*-Golgi [29]. The Interaction between SNAREs, in particular the v-SNARE SEC22B and the t-SNAREs, also brings the vesicle closer to cis-Golgi for fusion. COPII-coated vesicles fuse to the *cis*-Golgi through a regulated assembly of four tail-anchored transmembrane SNAREs, syntaxin 5, membrin, BET1 and SEC22B [42,43].

### 2.2. Vesicle Formation and Budding at the Golgi, and Fusion with the ER and within the Golgi

The Golgi apparatus is mainly involved in lipid and protein processing and their subsequent sorting to their final destinations. COPI-coated vesicles mediate retrograde trafficking (Figure 1), mostly of cargo receptors and SNAREs, from cis-Golgi back to the ER for reuse. The COPI coat is composed of a single heptamer consisting of the α-, β′-, ε-, β-, γ- and ξ-COP subunits, which are arranged into a cage-like outer sub-complex (α, β′- and ε-COP) and an adaptor-like inner sub-complex (β-, δ-, γ- and ξ-COP subunits) [44,45]. The inner sub-complex of the COPI coat mediates the sorting of cargo proteins into the vesicles [42]. The sequential assembly of this protein coat is initiated when small GTPases of the ADP-ribosylation factor (ARF) family are recruited and activated by the GEF GBF1 and SEC7 at the cis- and trans-Golgi, respectively [46]. Similar to the COPII coat, the COPI coat is formed by self-assembly of the inner coat elements, followed by the assembly of the outer cage [44,45]. The progressive multimerization drives the formation and budding of a COPI-coated vesicle. The release of the COPI coat from the vesicles is subsequently triggered by the GTP-activating protein (GAP) activity of the γ-COP subunit or possibly by ARF GAP2 [44,47,48].

ARF1, a member of the class I human ARF GTPase family, localizes to the Golgi apparatus and plays a central role in intra-Golgi vesicular trafficking by associating reversibly with phospholipids [49]. Through its ability to dimerize in its GTP-bound form and recruit actin, cortactin and dynamin 2, ARF1 is a key player in the biogenesis and budding of both COPI-coated vesicles and clathrin-mediated fission at the TGN [20]. These interactions suggest that ARF1-positive vesicles bud off from the Golgi membrane via the dynamin 2 GTPase activity and travel to their destination along actin filaments, facilitated by cortactin [20,50]. Thus, the cytoskeleton is actively involved in the release of COPI-coated vesicles from the Golgi.

The molecular details of the trafficking of vesicles between the Golgi and the ER is not fully understood, but it is known to involve a number of proteins, including motor protein kinesin, dynein, actin filaments and myosin V [51,52,53]. In retrograde trafficking, COPI-coated vesicles fuse with the subdomains of the ER, known as ER import/arrival sites (ERASs) [51,52,53] (Figure 1). ER-resident tethering factors, such as the *SLY1-20* (Dsl)/NAG-RINT1-ZW10 (NRZ) complex and the UVRAG protein (in mammals), ensure the specific binding of COPI-coated vesicles to the ER membrane and assist in the uncoating of the vesicles [51,54,55]. The recognition of ER is facilitated by the t-SNAREs, i.e., Ufe1, Sec20 and Use1 in yeast [51,54]. The Dsl1 subunit of the Dsl tethering complex binds to α-COP and δ-COP, while the Dsl3 and Tip20 subunits interact with Use1 and Ses20 at the ERAS [51,54]. ERASs are located in close proximity of the plasma membrane expansion hotspots, and they are highly rich in actin and myosin V [51].

At the TGN, proteins are sorted based on their interaction with specific receptors and/or vesicle coats and delivered to the plasma membrane or the endolysosomal system (Figure 1). Clathrin-mediated fission at the TGN, which also involves the adaptor protein complex 1 (AP-1) and/or the Golgi-localizing, γ-adaptin ear homology, ARF-binding protein (GGA) clathrin adaptors, is central for the sorting of proteins that are delivered to the endolysosomal compartments [18,20]. In contrast, the TGN machineries involved in the formation of vesicles directed to the plasma membrane, i.e., secretory vesicles, remain to be fully understood [18,20]. A significant number of secreted proteins are released from the cell either in a known coatomer, such as clathrin coats, or constitutively [18]. For example, immunoglobulins are secreted in smooth vesicles [20]. Vesicles without a protein coat or with a protein coat that is not yet identified also deliver membranes to the plasma membrane [18,20]. Nonetheless, it has been shown that RAB6 regulates vesicle fission at *trans*-Golgi [20]. Pull-down and microscopy experiments revealed that this fission is facilitated by a number of RAB6-interacting components, including motor protein kinesin-1 (KIF5B), dynein and myosin II, which interact with microtubules and F-actin filaments for fission of these vesicles at the TGN [20,53]. RAB6 is also important in delivering secretory vesicles to the plasma membrane. Here, the RAB6 functions along with anterograde cargoes, such as CD59, TNFα and ColX [20].

### 2.3. Vesicle Fusion at the Plasma Membrane and Exocytosis

A model termed ‘bulk flow’ proposes that secretory vesicles are sorted at the TGN and delivered to a specific region of the plasma membrane [56]. Cargo proteins of these secretory vesicles appear to not require a sorting signal for secretion, contrary to proteins transported to the endolysosomal system, and, therefore, their delivery happens in ‘bulk’ by default [20]. For example, ER-derived proteins transported to the Golgi by p24 or ERGIC53/LMAN1 receptors are subsequently secreted with no other known signal [20].

An important possible mechanism to promote the transport of cargoes from the Golgi to the plasma membrane is their preferential distribution into sphingomyelin-rich membranes, which are a constituent of secretory vesicles. Sphingomyelin is synthesized in the Golgi and concentrates at the plasma membrane [20,57]. Proteins, such as glycophosphatidylinositol-anchored proteins and CAB45 (and its binding partners, such as lysozyme C and insulin), are transported to the plasma membrane in sphingomyelin-rich vesicles [20,57,58]. The formation of these vesicles involves the activation of the secretory pathway calcium ATPase 1 (SPCA1) in the Golgi by interacting with cofilin-1 and F-actin, which pump calcium into the TGN for the oligomerization of soluble CAB45 and subsequent sorting of secretory carriers [58,59,60,61]. The small GTPase SEC4 tethers secretory vesicles to the exocytic SNAREs at the plasma membrane by interacting with SEC3 and SEC15 proteins, which are components of the exocyst complex [51]. Thus, SEC4 and the exocyst complex reside in the region of the plasma membrane where secretory vesicles are docked [51]. SEC3 and SEC15, among others, mark the sites of exocytosis at the plasma membrane for the appropriate and specific fusion of secretory vesicles [14,62,63]. The exocyst is a two-sub-complex molecule composed of eight subunits: SEC3, SEC5, SEC6 and SEC8 (sub-complex 1) and SEC10, SEC15, EXO70 and EXO84 (sub-complex 2). It is involved in the tethering of secretory vesicles to the plasma membrane, which is then followed by SNARE-mediated fusion [64]. RAB GTPases, such as the members of the SM protein family and the exocyst, control the cellular assembly of v-SNARE–t-SNARE complexes [34]. There are several RAB proteins associated with the secretion of vesicles, including RAB3, RAB7, RAB8, RAB10, RAB11, RAB12, RAB14 and RAB35 [14]. The cytoskeleton, particularly actin, also has an integral role in regulating exocytosis [51].

MUNC13-like proteins are other crucial molecules for the priming of vesicles at the plasma membrane, and along with Ca^2+^ sensor synaptotagmin-1, they potentiate the docking and exocytosis of secretory vesicles [29,65]. For example, the synaptic MUNC13-1 regulates the SNARE complex assembly and determines the priming of synaptic vesicles at the plasma membrane [66]. During fusion at the plasma membrane, the v-SNARE protein VAMP2/synaptobrevin II interacts with the t-SNAREs SYNTAXIN1 and SNAP25B [67]. Moreover, it has been shown, albeit in the context of insulin secretion from pancreatic β-cells, that increased intracellular Ca^2+^ raises the cytoplasmic ATP/ADP ratio and closes the K^+^ channels. This in turn results in the depolarization of the plasma membrane and Ca^2+^ influx and triggers exocytosis [31,68].

## 3. Formation and Functions of Viral-Induced Membrane Rearrangements

Although viruses are considered very simple organisms, consisting primarily of the viral genome wrapped in a protein and/or membrane shell, their replication cycles are relatively complex and diverse. In general, DNA viruses and retroviruses replicate and transcribe their genome in the host nucleus. By contrast, most RNA viruses carry out these processes in the cytoplasm. However, in both cases, viruses depend on cellular machinery, which they usurp and exploit not only for their cell entry, replication, transcription, assembly and egress but also for immune evasion [8,69,70]. Most RNA viruses and some DNA viruses, including members of the *Poxviridae*, *Parvoviridae* and *Herpesviridae* families, generate specialized compartments referred to as viral replication organelles, viral factories or viroplasm. These viral-induced structures solely benefit the virus and interfere with the cellular transcription, translation and secretion processes [71].

The ER and Golgi coordinate most intracellular transport networks. For successful infection, numerous viruses target the ER and the Golgi to exploit local cellular machineries to generate vesiculo-tubular membrane rearrangements and viral-induced vesicles [8,21]. These membrane rearrangements and vesicles are often essential for viral replication, serving as scaffolds for anchoring viral replication complexes, and virion morphogenesis, assembly and egress [8,18,21,69,72,73,74,75]. They can also prevent immune recognition of the RNA intermediates, tether viral RNA during unwinding and/or provide specific lipids required for genome synthesis and viral particle morphogenesis [8,21]. Despite being derived primarily from the ER, these viral-induced membranes can contain elements from endosomes (e.g., herpesviruses) [76,77], mitochondria (e.g., flaviviruses) [69,73,78], lipid droplets (LDs) (e.g., picornaviruses) [66,79] and other cellular compartments [18,80]. The presence of these elements indicates that crosstalk between different intracellular compartments is essential for the formation and/or maintenance of these virus-induced membrane rearrangements.

The exact mechanisms by which viruses induce the formation of membranous rearrangements from intracellular organelles remain largely unclear. However, these viral factories are thought to be formed by (a) the accumulation of large quantities of viral proteins that are produced in excess, (b) the targeting of viral proteins to specific cellular compartments and/or (c) the reprogramming of cellular aggresomes in order to concentrate structural components around the microtubule organizing center [73]. In addition, their formation often involves the recruitment and the regulation of factors involved in cellular processes, such as lipid biosynthesis and vesicular trafficking [81]. Other mechanisms mediating the formation of these structures include the rearrangement of the cytoskeleton and the reorganization and recruitment of specific organelles [8].

Two types of membrane modifications associated with viral infection have been identified and characterized. *Picornaviridae, Coronaviridae, Flaviviridae, Poxviridae* and *Herpesviridae* families all lead to the formation of cytoplasmic clusters of vesiculo-tubular membranes, which also include double-membrane vesicles (DMVs) [69,81,82,83]. DMVs are structures formed by clustering structural elements in either the nucleus or the cytoplasm of most viral-infected cells [69]. These vesicles are generally characterized by paired membranes, they have diameters between ~100 and 300 nm, and they are commonly associated with the replication of viral genomes [69]. The second type of membrane rearrangement formed by viruses during infection is the spherule invaginations, and they are generated by viruses belonging to *Flaviviridae*, *Coronaviridae*, *Togaviridae*, *Bromoviridae* and *Nodaviridae* families [69,81,82,84]. Spherule invaginations are formed by the inward curvature of the limiting membrane of intracellular organelles, such as the ER, mitochondria, endosomes and/or lysosomes [69]. A narrow channel of approximately 10 nm wide coordinates the movement of metabolites and viral molecules in and out of these spherule invaginations [69,78].

Viral factories are built in the perinuclear space/cytoplasm (e.g., coronaviruses) or in both the nucleus and cytoplasm (e.g., herpesviruses) [7,73]. Little is known about nuclear factories due to the limited knowledge about the nuclear sub-organization [7]. By contrast, cytosolic factories have been extensively studied, especially in the context of RNA virus infections [7,69,73,78,85,86]. These factories are often associated with the replication and intracellular trafficking of newly formed virions. Viral factories formed by DNA viruses, such as poxviruses, are also believed to be involved in virus replication [8,73,80]. Interestingly, viruses such as herpesviruses, which replicate in the nucleus, also appear to produce these cytoplasmic vesicle-like structures [77], which may serve as the sites for the assembly of new virions [77]. Some of the viral-induced membrane rearrangements are modified to produce viral envelopes (e.g., poxviruses) [73,76,87,88] or are required for tegumentation of the viral capsids (e.g., HSV-1) [72,75,85,86]. Despite the differences between viruses in creating these ultrastructures, some principles are similar [89]. Here, we discuss the general mechanisms with some examples.

### 3.1. RNA Viruses

#### 3.1.1. Picornaviruses

**Figure 2:**
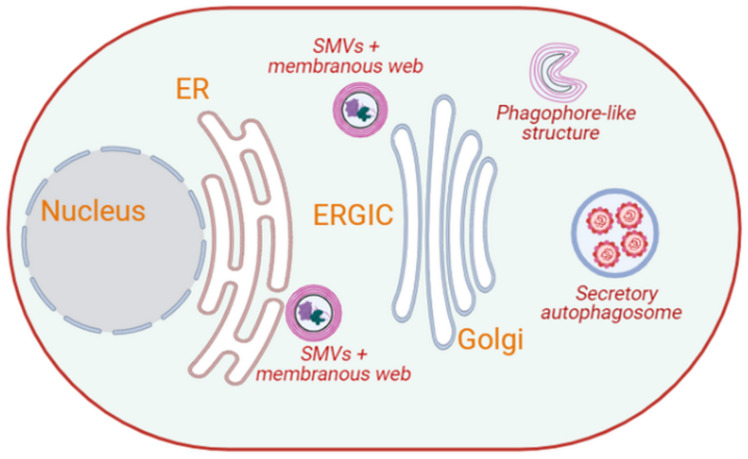
Intracellular membrane rearrangements induced by picornaviruses. Picornaviruses, such as poliovirus and CVB3, induce the formation of SMVs that contain non-structural proteins and dsRNA, and are embedded in a membranous web located adjacent to the ER. Over the course of the infection, these membranous webs relocalize near the Golgi, where crescent-shaped phagophore-like structures emerge from them. These phagophore-like structures may serve as the precursors to double-membrane autophagosomes, which appear approximately 6 hr post-infection. Complete picornaviral particles appear to exit cells using secretory autophagy. (Image created in Biorender).

Picornaviruses are a group of small RNA viruses that cause a wide range of diseases in humans and animals. They are non-enveloped viruses with a single stranded positive-sense RNA (+ssRNA) genome of ~7–9 kb and an icosahedral capsid with a diameter of around 30 nm [90]. The picornavirus genome encodes a single polyprotein that is cleaved to produce four structural and seven non-structural proteins [91]. As with other RNA viruses, picornaviruses replicate and assemble in the cytoplasm. Picornaviruses, such as poliovirus and coxsackievirus B3 (CVB3), induce the formation of single-membrane vesicles (SMVs) with a diameter of around 50–400 nm within a couple of hours post-infection [71,92]. These SMVs contain non-structural proteins and double-stranded RNA (dsRNA), and they are embedded in a matrix called the membranous web [81], which is associated with viral replication and virion assembly (Figure 2) [71,81,93,94]. They localize around the ER during the early phases of the infection [71,95] and redistribute near the *cis*-Golgi when the infection progresses [92]. Later in the infection, the membrane of these vesicles form convoluted invaginations, making them look like crescent-shaped cisterns from which DMVs may emerge (Figure 2) [92,96].

Replication of the genome of poliovirus interferes with the cellular secretory pathway by inhibiting the members of the ARF GTPase family [97]. Both poliovirus and CVB3, and possibly other members of the picornavirus family, recruit ARF GTPases and their activating GEF to the sites of RNA replication [98,99]. Moreover, the non-structural proteins of poliovirus predominantly co-localize with components of COPII-coated vesicle formation machinery at the SMVs [95,97]. However, there is evidence that replication may also occur at the DMVs [96]. In addition to the COPII-coat components, the lipid kinase phosphatidylinositol 4-kinase IIIβ (PI4KIIIβ) is also essential for CVB3 replication at the SMVs [79].

Both PI4KIIIα and PI4KIIIβ are implicated in the process by which viruses hijack the cellular secretory pathway [18]. Class III phosphatidylinositol 4-kinases are Golgi lipid kinases and are enzymes that define the structure of the Golgi and TGN. They regulate the trafficking-associated functions in these compartments. PI4KIIIα/β increases the level of intracellular phosphatidylinositol 4-phosphate (PI4P) [18,19,100,101]. Enteroviral membrane proteins, such as 3A of CVB3, have been shown to recruit PI4KIIIβ via the ARF1-specific GEF GBF1 to the viral replication SMVs [70,79,98,102]. PI4KIIIβ, however, can also be recruited to these vesicles in an ARF1-GEF GBF1-independent manner [79]. The co-localization of the ARF1 GTPase and the PI4KIIIβ in a replication vesicle produces a PI4P lipid-enriched microenvironment [98]. The accumulation of PI4P-rich lipid within this microenvironment is essential for viral replication because it facilitates the recruitment of RNA-dependent RNA polymerase 3D^pol^, along with other viral and host proteins needed for the replication [18,98,102].

The synthesis of poliovirus RNA is affected when autophagy is non-specifically inhibited by 3-methyladenine [103]. Similarly, when autophagy is altered by inhibiting the acidification of cellular compartments, poliovirus maturation is affected by up to 90%, leading to a decrease in the production of infectious virus particles [103]. Both poliovirus and CVB3 assembly vesicles are believed to be DMVs derived from SMVs, which are redistributed near the *cis*-Golgi when the infection progresses [8,81,98]. Interestingly, DMVs formed by these viruses carry the autophagy marker proteins BECLIN1 and LC3, and, therefore, they are also referred to as autophagosome-like vesicles [8]. These vesicles enhance the replication, assembly and egression of these viruses. They fuse with the plasma membrane through a process that involves the cytoskeleton and secretory autophagy (Figure 2) [104,105,106]. Autophagosomes interact with compartments of the endolysosomal system, such as MVBs, to generate amphisomes that fuse with lysosomes to promote the degradation of their contents [107]. MVBs are important components of the endocytic pathway and contribute to autophagy [107]. MVBs are also known to transport their contents to the plasma membrane [107,108]. Therefore, the egression of poliovirus may be linked to the MVBs/exosome secretion system [8].

#### 3.1.2. Coronaviruses

Coronaviruses are a large group of enveloped +ssRNA viruses that infect both humans and animals [109,110]. They are known to cause respiratory illnesses with mild-to-severe symptoms. The large genome of coronaviruses (26–32 kb) encodes for four structural and 14–16 non-structural proteins on a single polypeptide [110]. Coronaviruses are grouped into four genera: α, β, γ and δ [110,111]. Currently, seven members are known to infect humans, five of which have been isolated since 2003, with the most recent one being severe acute respiratory syndrome coronavirus-2 (SARS-CoV-2), the cause of the current COVID-19 pandemic [112].

**Figure 3:**
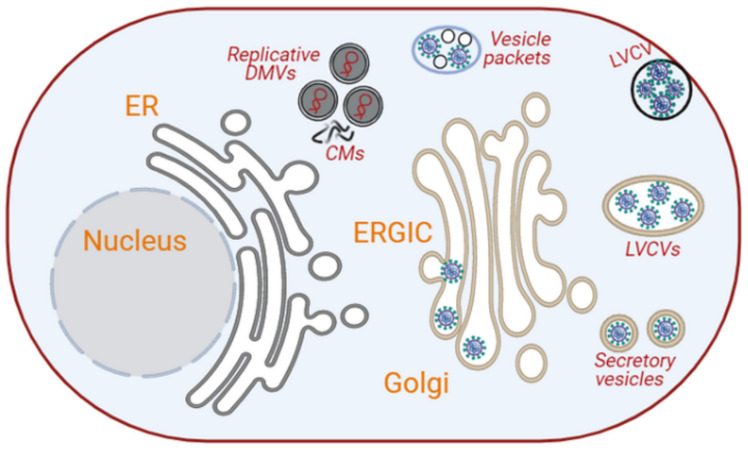
Intracellular membrane rearrangements induced by coronaviruses. Coronaviruses induce the formation of branched and unbranched ER vesicles, DMVs and CMs from the ER. The DMVs serve as platforms for replication/transcription. Initially, coronaviral particles assemble at the ERGIC/Golgi, which expand into LVCVs to accommodate more virion productions (image created in Biorender).

Coronaviruses have been shown to induce the formation of three different types of membrane rearrangements that are connected with viral replication and transcription. These include (i) regular cytoplasmic DMVs of 200–300 nm in diameter; (ii) convoluted membranes (CMs) or zippered ER, which would represent branched or unbranched configurations of paired ER membranes; and (iii) small open double-membrane spherules (DMSs) that appear to arise from the CMs (Figure 3) [84,85,86]. In between these three membrane rearrangements, DMVs are the ones that support viral replication [69,83,86]. Their outer membranes are directly or indirectly linked to the ER though the convoluted membranes (CMs) [69,81,84]. Recently, it has been shown that DMVs are not close compartments but possess a molecular pore complex, in which a hexamer formed by the large viral transmembrane nonstructural protein 3 generates a crown-shaped core, spanning both membranes of the DMVs [113]. This finding underscores a model in which viral RNA synthesis occurs in the lumen of the DMVs (Figure 3), and the molecular pore complex would allow the export of RNA to the cytosol for translation or encapsulation into progeny virions [113].

Soon after infection, the positive-sense RNA genome of coronaviruses is translated into 14–16 non-structural proteins [111,114,115]. These proteins are responsible for the formation of the DMVs from the ER into which the replication–transcription complexes (RTCs) are anchored [115,116]. The formation of DMVs has been observed as early as 2 h post-infection [86]. The early DMVs have sizes ranging from 150 to 300 nm; they are distributed throughout the cytoplasm, and they are occasionally connected to small CMs, which are also positive for the RTC components [86]. At about 3 h post-infection, the DMVs and CMs associate with some large structures of 0.2–2 µm, which resemble reticular inclusions, and are connected to the ERGIC [86]. As the infection progresses, the number of DMVs and the size of the CMs increase, they concentrate in the perinuclear area of the cell, and their connection with reticular membrane structures becomes clearer [85,86,115]. At what stage during the course of coronavirus infection DMSs appear, and what their function is, remains unclear. Since DMSs are connected with the CMs, one speculation is that they contain components of the RTCs [85]. These membrane rearrangements may, at least in part, be generated by the hijacking of the ER-associated degradation (ERAD) pathway, a direct transport route from the ER to the compartment of the endosomal systems involved in the turnover of ERAD regulators, such as EDEM1 and OS-9 [117]. The translocon component SEC61α has also been detected on membrane rearrangements induced by SARS-CoV-1 [118], but the relevance of this protein for coronavirus replication remains to be established.

The initial assembly and luminal budding sites of coronaviruses are the ERGIC and Golgi. The transmembrane structural proteins are translocated into the ER, and from there, they reach these compartments by vesicular transport where interaction with the soluble nucleocapsid protein loaded with genomic RNA triggers the inward budding of viral particles. These compartments can accommodate either a single virion or multiple virions (Figure 3) [115,119,120]. Over the course of the infection, these Golgi compartments expand to accommodate the increased synthesis of structural proteins and virion assembly, leading to the formation of the so-called large virion-containing vacuoles (LVCVs) (Figure 3) [115]. These LVCVs have been observed during SARS-CoV-1 and SARS-CoV-2 infections [86,120]. LVCVs appear to be able to release virions not only through fusion with the plasma membrane but also via the formation of tunnels between them and the plasma membrane [120].

A recent investigation revealed that coronaviruses from the β-genus can assemble in the ER [121], something that was reported to occur at later stages of β-coronavirus infection [115] and for viruses belonging to the γ-genus [122]. This study also suggested that virions may be transported from the ER to de-acidified lysosomes, probably through an autophagy-related pathway and subsequently secreted by lysosome fusion with the plasma membrane [121].

#### 3.1.3. Flaviviruses

Flaviviruses are enveloped RNA viruses with a total of 58 species classified into 3 genera, namely Flavivirus, Hepacivirus and Pestivirus [123]. The genome encodes a single polyprotein, which is processed by viral and cellular proteases into three structural and seven non-structural proteins. This family includes many disease-causing viruses, including the hepatitis C virus (HCV), which belongs to the genus Hepacivirus. HCV is associated with liver inflammation, fibrosis, cirrhosis and hepatocellular carcinoma [124]. Other human pathogenic viruses, such as dengue virus (DENV), Zika virus (ZIKV) and West Nile virus (WNV), are classified within the Flavivirus genera and are transmitted by mosquitoes. These viruses result in a wide range of symptoms ranging from mild febrile illness to severe disease, such as dengue shock syndrome, hemorrhagic fever, Guillain–Barré syndrome and encephalitis [125].

**Figure 4:**
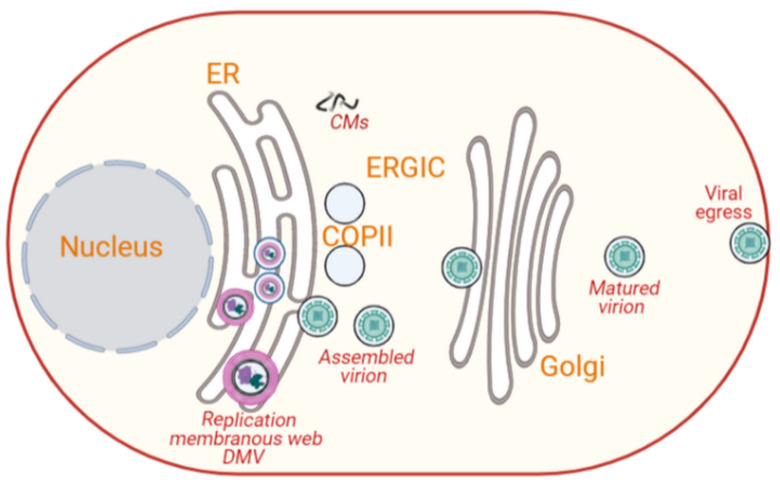
Intracellular membrane rearrangements induced by flaviviruses. Flaviviruses, such as HCV, lead to production of DMVs through the curvature of the ER membrane. These DMVs are embedded into a membranous web and carry non-structural proteins, and they are positive for dsRNA. Flaviviruses assemble close to the ER and acquire their envelope by budding into ER, and they usurp specific ER functions, such as the membrane trafficking apparatus, for their intracellular trafficking to the plasma membrane via the Golgi for final exit (image created in Biorender).

The examination of cells infected by HCV show the presence of DMVs encapsulated in a membranous web (Figure 4) [81]. DMVs produced by HCV are similar to those formed by picornaviruses in that they are embedded into a membranous web, carry non-structural proteins and dsRNA, form clusters and are associated with viral replication [81,93,94]. However, the DMVs produced by HCV are smaller [81]. At about 16 h post HCV infection, DMVs of ~125 nm are observed, and their appearance correlates with an increase in viral RNA replication [80,81]. The HCV non-structural protein NS4B has been implicated in the formation of these DMVs [126]. Similar to picornaviruses, the formation and maintenance of the membranous web during HCV infection is governed through the generation of PI4P by PI4KIIIα. The highly negative-charged head group of PI4P leads to ER membrane curvature [100,127]. Furthermore, PI4P recruits lipid transfer proteins, such as oxysterol-binding protein (OSBP), which have been implicated in HCV replication [128]. Similar to CVB3 NS3A, HCV NS5A recruits ARF1, GBF1 and PI4KIIIβ from the cellular secretory pathway organelles of one these replicative factories to generate them [70,98,102]. While ARF1 and GBF1 are dispensable for CVB3, they appear to be essential for HCV infection [98]. Other members of flaviviruses, such as DENV and WNV, also form spherule-like invaginations and CM derived from local proliferation of the ER membrane and represent the site of replication [78,89].

Surprisingly, PI4KIIIα is not involved in the viral cycle of some flaviviruses, such as DENV [129], but interestingly, it has been shown that WNV and DENV require fatty acid synthase for replication [130]. As members of flaviviruses, such as HCV, DENV and ZIKV, encode polyprotein with an ER localization signal sequence [10,22,23], it is not surprising that these viruses usurp specific ER functions, including the protein translocation machinery, signal peptide processing system, *N*-linked glycosylation and the membrane trafficking apparatus, to exit the ER [131,132,133,134]. For example, a recent CRISPR screen revealed that members of the *Flavivirus* genus hijack the SEC61 translocon complex, via the SEC61 and SEC63 subunits, and several components of the translocon-associated protein (TRAP) complex (composed of SSR1, SSR2, SSR3, RPL31 and TRAM1). Similar to the host secretory proteins, SEC61-mediated cotranslational translocation into the ER is also essential for the biosynthesis of viral proteins and, ultimately, viral particles [131,132,133]. Moreover, it appears that DENV replication is severely impacted in cells deficient in the oligosaccharyltransferase complex, highlighting the importance of *N*-linked glycosylation [133]. This exploitation of the ER machinery is essential to promote several steps of the viral life cycle [131,132,133]. For example, proper cleavage of the flaviviral M and E structural proteins, as well as the viral egress, depend on the ER-associated signal peptidase complex (SPCS) proteins [135]. Evidence for this has also been provided for the DENV glycoproteins [134]. Finally, flaviviruses assemble close to the ER, and most flaviviruses acquire their envelope by budding out of the ER (Figure 4) [18]. Final maturation is carried out while the virus particles are transported to and through the Golgi until they exit the cell. Since HCV uses SAR1A, it is thought that the transport from ER to Golgi occurs using COPII vesicles [136]. Another important step in flavivirus maturation, which is well characterized for DENV, is the proteolytic cleavage of the prM viral protein by host protease furin in the TGN. The furin cleavage is crucial for converting immature virus particles into fully mature virus particles [137,138].

Flaviviruses also remodel the cytoskeleton and other cellular organelles, including mitochondria, LDs and autophagosomes [69,78]. The cytoskeleton, in particular the intermediate filament and the microtubules, is known to be reorganized upon infection with DENV. The intermediate filament protein vimentin has been shown to interact with DENV NS4A, enabling perinuclear localization of the replication complex [139,140]. LDs, which are located in close proximity to the replication sites, support virus assembly during HCV infection. Here, the virus-encoded core and NS5A are targeted to produce infectious virions [81,93,141,142]. Moreover, DENV is known to usurp autophagy to selectively target LDs to modulate their catabolism and support virus replication through the generation of energy [143]. Flaviviruses also use ESCRT machinery for virus particle budding and envelop acquisition [144,145].

### 3.2. DNA Viruses

Some DNA viruses also induce membrane rearrangements and vesicle formation in host cells. These are created in both the nucleus and/or cytoplasm. Moreover, an empty area devoid of any cellular proteins or organelles has been observed near the nucleus of cells infected with nuclear-replicating DNA viruses, such as herpesviruses [73]. This area marks the site where viral factories are formed [73]. Vesicle-like structures have also been seen in the cytoplasm of cells infected with DNA viruses, such as those from the *Poxviridae*, *Parvoviridae* and *Herpesviridae* families. These structures are rich in viral structural proteins and DNA, suggesting they could mediate viral particle assembly [7,31,68]. Similar to RNA viruses, mitochondria and cytoskeleton have also been associated with the cytoplasmic vesicle-like structures formed by specific DNA viruses [7,31,68]. These organelles may help in creating contacts and communication between the virus-induced vesicles and other cellular compartments but also facilitate viral egression [7,73].

#### 3.2.1. Poxviruses

**Figure 5:**
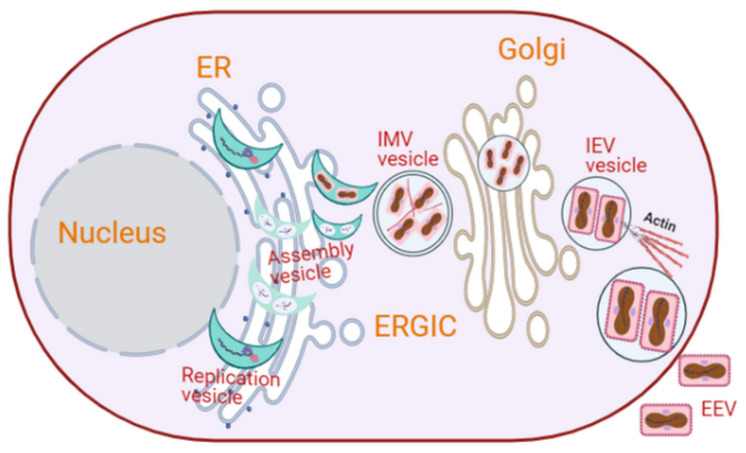
Intracellular membrane rearrangements induced by poxviruses. Vaccinia virus replicates in cytoplasmic crescent-shaped vesicle-like structures, resembling mini-nuclei. An intact membrane is observed around the replication organelle during DNA replication but disappears at the initiation of viral assembly. Vimentin is recruited to the replication organelle, and it facilitates virus assembly. This assembly gives rise to infectious IMVs in a double-membrane cisterna derived from the smooth ER, which later acquire single lipid membranes from the ERGIC and are subsequently wrapped by the TGN to form an IEV. While most of the vaccinia exits host cells by cell lysis, a small proportion of the IEV polymerizes actin tails, and they are released extracellularly as an EEV (image created in Biorender).

Poxviruses are large, complex, enveloped double-stranded DNA viruses surrounded by a capsid with a diameter of around 300 nm, which infect both humans and animals. Poxviruses are unique DNA viruses because they replicate and complete their life cycle in viral-induced subcellular structures in the cytoplasm [9,73,88]. Poxviridae family is subdivided into subgroups: Entomopoxvirinae and Chordopoxvirinae [146]. Vaccinia virus, the source of the smallpox vaccine, is one of the best-characterized members of the Poxviridae family. Its genome is approximately 190 kb and encodes more than 200 proteins, and about 100 of them are incorporated into the virion [73,147]. Vaccinia virus replicates in cytoplasmic vesicle-like structures, resembling mini-nuclei, which originate from the ER (Figure 5) [8,73,146]. The viral cycle of vaccinia virus takes place in several cytoplasmic compartments [74]. Viral and cellular factors shuttle between these vaccinia virus-induced structures and the host cytoplasm [74]. The life cycle of vaccinia virus starts at the plasma membrane soon after infection [74]. Within 20 min of infection, the early genes of vaccinia virus are translated into proteins, including E8R, a protein that is believed to mediate the creation of cytoplasmic replication of vesicle-like structures [74]. E8R is integrated into the ER and surrounds the viral DNA [73,74]. At 2 h post-infection, cytoplasmic membranes with typical crescent-shaped structures that serve as replication sites for the virus begin to appear (Figure 5) [74].

The maturation of vaccinia virions in these vesicle-like structures shares several analogies with the nuclear envelope assembly/disassembly during cell cycle. That is, the ER membrane that makes the outer layer of the vesicle-like structures of vaccinia virus during its DNA replication is dispersed when virion assembly begins, i.e., after replication is ceased [73,74]. This phenomenon is similar to nuclear membrane disassembly and reassembly during mitosis and late anaphase/telophase. An intact nuclear membrane is essential for cellular DNA replication [74]. The vesicle-like structures involved in vaccinia virus replication remain visible for up to 6 h post-infection when virion assembly is initiated [74]. During the vaccinia virus assembly, vimentin, an intermediate filament essential for the cytoskeleton dynamics, is recruited to the vaccinia-induced vesicle-like structures to facilitate virus assembly by incorporating viral proteins into the assembling particles [73]. In a related study, it was shown that cellular aggresomes surrounded by vimentin also facilitate viral assembly [9,12]. At this point, a double-membrane cisterna derived from the smooth ER appears around the viral core, giving rise to intracellular mature viruses (IMVs) that are infectious (Figure 5) [74,148]. More recent studies have reported that the IMVs mature by acquiring single lipid membranes derived from the ERGIC [149,150]. A small proportion of the IMVs get wrapped by the TGN and acquire a primary envelope, leading it to metamorphose into an intracellular enveloped virus (IEV) [74,151,152]. An IEV polymerizes actin tails to be released as an extracellular enveloped virus (EEV) (Figure 5) [74,152]. However, this is a minor egression mechanism. Most vaccinia viral particles exit host cells by cell lysis, which release IMVs.

During vaccinia virus infection, mitochondria distribute and concentrate proximal to the cytoplasmic virus-induced vesicles localizing to the perinuclear area and around the Golgi. These locations correspond to the sites of the virus replication, assembly and maturation [73]. This redistribution is thought to be mediated by the cytoskeleton, such as microtubules and the microtubule-organizing center [7,9]. The fact that the mitochondria regulate many critical cellular processes, including energy production and Ca^2+^ signaling [153,154], possibly implies that there is an involvement of intracellular Ca^2+^ signaling pathways in the exocytosis of vesicles containing mature vaccinia virions [31,59,60,61,68].

#### 3.2.2. Parvovirus

**Figure 6:**
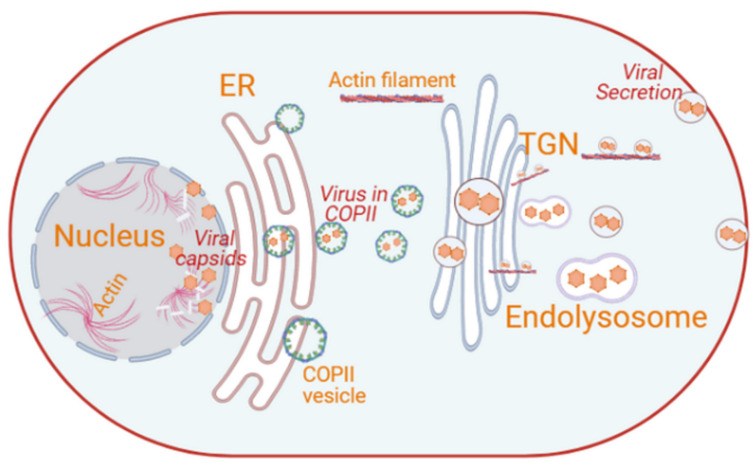
Intracellular membrane rearrangements induced by parvoviruses. Parvoviruses replicate and assemble their capsid in the nucleus. The nucleocapsid is transported from the nuclear periphery to the plasma membrane in a gelsolin-dependent manner. Cytoplasmic parvoviral particles are engulfed in COPII-coated vesicles at the ER, following its traffic to the Golgi. Matured virus particles are then transported to the plasma membrane possibly via the secretory pathway and actin filaments through gelsolin and are then released by activation or modulation of the PDK1/PKC/PKB signaling cascade (image created in Biorender).

Parvoviruses are small, non-enveloped lytic viruses with a linear single-stranded DNA of 5–6 kb and a capsid with a diameter of approximately 25 nm [155]. Parvoviruses infect both humans and animals. Infection in humans can be pathogenic or non-pathogenic. Parvovirus B19 and human bocavirus 1 are two human pathogens [155]. Parvoviruses replicate and assemble their capsid in the nucleus (Figure 6). The nucleocapsids are transported into the cytoplasm in a gelsolin-dependent manner [156,157]. Gelsolin is an actin-cleaving protein that plays a significant role in intracellular trafficking and the egress of parvoviruses [27]. Parvoviruses mature and gain full infectivity along their journey through the secretory pathway [27]. Parvovirus non-structural protein NS1 interacts with the catalytic domain of the cellular casein kinase II to form the NS1/CKIIα complex. This complex controls both gelsolin-dependent actin degradation and the release of progeny virions into the extracellular space [87,156,158]. Upon associating with the ER, parvoviral nucleocapsids are engulfed into COPII-coated vesicles for trafficking in an anterograde manner to the Golgi (Figure 6) [27]. This is underlined by the colocalization of parvoviral capsids with ER-localized calnexin and the components of COPII-coated vesicles, such as SEC24, SEC13 and SEC23 [27]. These COPII-coat components and associated factors, such as SAR1 and RAB1, and two members of the ezrin protein family, radixin and moesin (ERM), appear to be essential in the formation of parvovirus-containing COPII-coated vesicles [27]. ERM proteins are cellular proteins that mediate the interaction between filamentous actin and cellular membrane structures [27].

Additionally, parvoviral particles, detected using antibodies against the viral capsid, colocalize with Golgi-resident marker proteins GM130 and β-COP; the TGN GTPases RAB6, RAB1 and RAB11; and the lysosomal protein LAMP2 [27,156], thus indicating the implication of the compartments of the secretory pathway in their egress from the cell. Parvoviral DNA and SEC23, radixin and moesin were detected in vesicular fractions released by parvovirus-infected cells [27]. Treating parvoviral-infected cells with dominant-negative moesin (MoeT547A) and radixin (Rdxdl[P]) mutants resulted in low-level detection of capsids in the cytoplasm and subsequent inhibition of virion release into the medium [27].

The NS1/CKIIα complex also mediates the rearrangement and disassembly of both the cytoskeletal micro and intermediate actin filaments but not the microtubule network. Therefore, the kinase activity of CKIIα could also be important in the parvoviral ability of modulating the secretory system [27,159]. Radixin is one of the cellular proteins targeted by the NS1/CKIIα complex [27]. It allows parvoviral NS1 to indirectly activate/modulate PDK1/PKC/PKB signaling through the activation of radixin, which is an adaptor for the kinase PKCη [27,160]. PKCη is a Golgi-associated member of the protein kinase C (PKC) family that is involved in protein transport from the TGN to the plasma membrane, while PDK has been shown to be involved in membrane fission at the TGN [161]. Thus, the activation of the PDK1/PKC/PKB signaling cascade facilitates the fusion of the virus-containing vesicles with the plasma membrane, which is essential for their release in a gelsolin-dependent manner (Figure 6) [27,87,156].

#### 3.2.3. Herpesviruses

**Figure 7:**
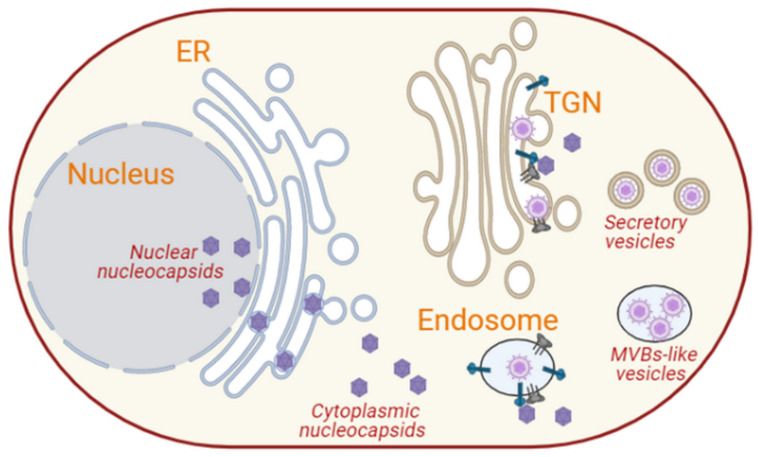
Intracellular membrane rearrangements induced by herpesviruses. Herpesviruses replicate and assemble their capsids in the nucleus, in which their glycoproteins are also targeted. They transiently acquire a primary envelope when crossing the inner nuclear membrane and lose it when exiting from the outer nuclear membrane. This passage also involves the disassembly of the nuclear lamina. Capsid and glycoproteins are transported from the ER to the TGN, where they interact with the cytoplasmic capsids to form intraluminal virions by inward budding. These viral particles are then transported to the plasma membrane and released extracellularly. Some herpesviruses, such as HHV-6, assemble their viral particles at the endosomes, generating MVB-like compartments that fuse with the plasma membrane to release the luminal virions (image created in Biorender).

Herpesviruses are large, enveloped, linear double-stranded DNA viruses, with icosahedral capsids and a diameter of around 125 nm surrounded by a layer of proteinaceous material called tegument [70,75,162]. Herpesviruses infect both humans and animals, causing symptoms such as cold sores, chicken pox and cancer [163,164]. After primary infection, they establish latency that often persists for the entire life of its host and may result in occasional reactivation when conditions are favorable. The genome of herpesviruses contains several dozen structural proteins distributed in three viral layers: capsid, tegument and envelope [162]. *Herpesviridae* are subdivided into three subfamilies, α-, β- and γ-*herpesvirinae*, based on their genomic sequence and biological parameters, including cytopathic effects [75]. Herpesviruses, similar to most other DNA viruses, replicate and assemble their nucleocapsids and glycoproteins inside the nucleus (Figure 7). An empty area devoid of any cellular proteins or organelles has been observed in the nucleus of infected cells near the spherical bodies referred to as the nuclear domain 10 (ND10) [73]. This area marks the site where viral replication factories are formed [73].

After replication and capsid assembly, nucleocapsids are transported from the nucleus to the cytoplasm. In contrast to the nucleocapsid of many other DNA viruses that pass through the nuclear pores or rupture the nuclear envelope for their nuclear egress [165], herpesviruses disassemble the nuclear lamina and gain their primary envelope as they pass through the inner lumen of the nuclear envelope [77,165,166,167]. Viral envelope glycoproteins are implicated in this passage, as they have been shown to be present at the inner nuclear membrane [77]. These glycoproteins, which are also present in the viral primary envelope, are believed to mediate fusion with the outer nuclear membrane to release the non-enveloped nucleocapsids into the cytosol [77,165,166]. Although little is known about this fusion step, one possible scenario is that the nucleocapsids interact with the glycoproteins present in the perinuclear space before the enveloped capsids fuse with the outer nuclear membrane to release membrane-less capsids into the cytoplasm. For example, it has been reported that the glycoproteins gB and gH of HSV-1 are required for crossing the nuclear envelope [168]. It has also been reported that the viral nucleocapsids and the glycoproteins are transported in the same vesicle through the cytoplasm to their site of assembly [162,169]. Other models suggest that the nucleocapsids and the glycoproteins are transported separately [169].

The cytoplasmic nucleocapsids and glycoprotein-containing vesicles/membranes are transported in a kinesin motor-dependent manner along microtubules to the Golgi/TGN for the final envelopment [77,162,169]. The gE/gI, US9, UL36p and UL37p proteins of HSV-1 have been shown to play a crucial role in mediating this interaction between the cytoplasmic nucleocapsids and glycoprotein-containing vesicles and microtubules [162,169]. At the TGN, the nucleocapsids and glycoprotein-containing membranes interact via the tegument to form luminal viral particles (Figure 7) [169]. There are two possible paradigms explaining how herpes viral particles are assembled. In the first model, the cytoplasmic nucleocapsids interact with the glycoproteins that have reached the TGN, and this event triggers the inward budding of the nucleocapsids, leading to the formation of mature virions [77,162]. Virions are then released in the extracellular milieu by fusion of the TGN/late endosome-derived virus-containing vesicles with the plasma membrane (Figure 7) [72,73,75,76,77,162,170,171,172]. In the second model, the glycoproteins that are endocytosed from the cell surface interact with the cytoplasmic nucleocapsids in the endosomes. This in turn leads to the intraluminal assembly of virions and their subsequent secretion via the fusion of the late endosomes with the plasma membrane (Figure 7) [162].

Phosphorylation, palmitoylation and myristoylation are crucial in the morphogenesis and maturation of herpesviruses [72]. The myristoylation of tegument proteins UL11 in HSV-1, UL99 of the CMV and BBLF1 of the Epstein–Barr virus (EBV) mediates the TGN membrane anchoring and stabilization for efficient interaction and incorporation of tegumented nucleocapsids into a glycoprotein-embedded membrane during the intraluminal viral particle budding [173]. Similarly, the palmitoylation of the tegument proteins also promotes proper membrane targeting and stabilizes membrane anchoring for viral particle assembly [173]. The cytoskeleton is also important in the assembly, maturation, trafficking and egress of herpesviruses. The tegument of HSV-1 was found to associate with short actin-like filaments, which then cluster viral glycoproteins and promote inward budding into the TGN [72,73]. Moreover, microtubules, actin filaments and focal adhesions contribute significantly in maintaining the structure of HSV-1 egress sites at the plasma membrane [27,72]. The depolymerization of actin during HSV-1 infection could promote the cellular egress of the viral particles by causing a depletion of the actin cortex and thereby creating holes that are persistent since herpesviruses also block actin repolymerization [170].

The actin cytoskeleton is also an essential component of the ERAS, and it also regulates the exocytosis of secretory vesicles at the plasma membrane expansion hotspots [51,135]. Thus, the depolymerization of actin could facilitate capsid movement in a retrograde manner as well as cell egress of the progeny virion. The HSV-1 proteins gE/gI and gB, along with host proteins, such as Golgi-localized TGN46 and lysosomal carboxypeptidase D, accumulate at the sites of cell–cell contact, and they interact with junctional components, such as cell adhesion molecules and cytoskeleton elements, to facilitate the egress and spread of the virus during reactivation [72].

Cells infected with HHV-6, a β-herpesvirus, trigger the formation of cytoplasmic MVB-like compartments (Figure 7). These MVB-like structures contain small vesicles that carry viral components, including the viral structural protein, gB and even mature virions [77], suggesting that these carriers serve as sites of HHV-6 assembly and maturation. These structures thus make the infected T-cells appear larger than uninfected T-cells [77]. The MVB-like structures surround the Golgi apparatus and express endosomal marker proteins, such as CD68 and clathrin, indicating that they may originate from endosomes [77]. These virus-containing MVBs fuse with the plasma membrane to release the virus through exocytosis (Figure 7) [77]. Thus, the assembly and cellular egress of HHV-6 may take place through a mechanism different from the one described above and in which the nucleocapsids interact with glycoproteins at the endosomes (Figure 7) [162]. Virions assembled by inward budding at the endosomes are then secreted via the fusion of these MVB-like late endosomes with the plasma membrane (Figure 7) [162]. The two models for herpesvirus egression may not be mutually exclusive but just describe two cell egression modes adopted by different members of the herpesvirus family.

Gamma herpesviruses, such as EBV, induce the formation of cytoplasmic compartments that vary in size and contain one or more enveloped capsids with spike-like protrusions and tegument material [76]. This indicates that they could be sites for viral maturation. However, unlike other herpesviruses, such as HHV-6, EBV structural proteins do not prominently co-localize with endosomal marker proteins, such as CD63 and RAB11, and secretory vesicle marker proteins, such as RAB27a [76]. The EBV envelope glycoprotein gp350/220 and viral capsid antigen co-localize with cis-Golgi and TGN proteins, GM130 and TGN46, respectively [76]. This co-localization suggests that the cytoplasmic compartments for the final envelopment and maturation of EBV are derived from the Golgi apparatus (Figure 7). Accordingly, these compartments have been detected in the vicinity of the plasma membrane and adjacent to Golgi-derived clathrin-coated vesicles, prompting the possibility that virion egress takes place through exocytosis [76].

The EBV tegument protein BBLF1 is both myristoylated and palmitoylated and contains a tyrosine-based sorting signal, YXXΦ [173,174]. This signal is hypothesized to promote budding of the tegumented capsid into a glycoprotein-containing vesicle for virus maturation [173]. YXXΦ is present in the cytosolic domains of cellular transmembrane proteins and facilitates the sorting of proteins to several subcellular compartments, including clathrin-coated vesicles and the plasma membrane [173,174]. Thus, BBLF1 appears to play a key role in the production of mature viral particles. However, the details of this mechanism remain mostly unknown.

## 4. Conclusions

Viruses are obligate intracellular parasites, hijacking cellular machineries to promote their propagation. The intracellular transport and secretion of viral components is a highly complex process. Multiple factors and pathways appear to be involved. Although recent advances in microscopy and imaging technologies have contributed significantly to our understanding of the functions and the dynamics of the cellular secretory pathway during viral infections, much more remains to be elucidated. For example, how viral proteins are targeted to the ER and to specific transport vesicles is still unclear. Several steps of vesicular transport, such as docking and fusion of virus/viral protein-containing vesicles with their acceptor compartment and how they are released from the plasma membrane, are only partially understood. A detailed understanding of such mechanisms is essential not only for delineating viral life cycles and understanding their pathogenesis but also to design therapies aimed at preventing or treating viral infections.

## Data Availability

Not applicable.
